# Enteric virome of Ethiopian children participating in a clean water intervention trial

**DOI:** 10.1371/journal.pone.0202054

**Published:** 2018-08-16

**Authors:** Eda Altan, Kristen Aiemjoy, Tung G. Phan, Xutao Deng, Solomon Aragie, Zerihun Tadesse, Kelly E. Callahan, Jeremy Keenan, Eric Delwart

**Affiliations:** 1 Blood Systems Research Institute, San Francisco, California, United States of America; 2 University of California San Francisco, Department of Laboratory Medicine, San Francisco, California, United States of America; 3 Francis I. Proctor Foundation, University of California San Francisco, San Francisco, California, United States of America; 4 Department of Epidemiology and Biostatistics, University of California San Francisco, San Francisco, California, United States of America; 5 The Carter Center Ethiopia, Addis Ababa, Ethiopia; 6 The Carter Center, Atlanta, Georgia, United States of America; Oklahoma State University, UNITED STATES

## Abstract

**Background:**

The enteric viruses shed by different populations can be influenced by multiple factors including access to clean drinking water. We describe here the eukaryotic viral genomes in the feces of Ethiopian children participating in a clean water intervention trial.

**Methodology/principal findings:**

Fecal samples from 269 children with a mean age of 2.7 years were collected from 14 villages in the Amhara region of Ethiopia, half of which received a new hand-dug water well. Feces from these villages were then analyzed in 29 sample pools using viral metagenomics. A total of 127 different viruses belonging to 3 RNA and 3 DNA viral families were detected. *Picornaviridae* family sequence reads were the most commonly found, originating from 14 enterovirus and 6 parechovirus genotypes plus multiple members of four other picornavirus genera (cosaviruses, saliviruses, kobuviruses, and hepatoviruses). Picornaviruses with nearly identical capsid VP1 were detected in different pools reflecting recent spread of these viral strains. Next in read frequencies and positive pools were sequences from the *Caliciviridae* family including noroviruses GI and GII and sapoviruses. DNA viruses from multiple genera of the *Parvoviridae* family were detected (bocaviruses 1–4, bufavirus 3, and dependoparvoviruses), together with four species of adenoviruses and common anelloviruses shedding. RNA in the order *Picornavirales* and CRESS-DNA viral genomes, possibly originating from intestinal parasites or dietary sources, were also characterized. No significant difference was observed between the number of mammalian viruses shed from children from villages with and without a new water well.

**Conclusions:**

We describe an approach to estimate the efficacy of potentially virus transmission-reducing interventions and the first complete (DNA and RNA viruses) description of the enteric viromes of East African children. A wide diversity of human enteric viruses was found in both intervention and control groups. Mammalian enteric virome diversity was not reduced in children from villages with a new water well. This population-based sampling also provides a baseline of the enteric viruses present in Northern Ethiopia against which to compare future viromes.

## Introduction

Limited access to clean drinking water is an enduring health hazard that can exacerbate enteric and malnutrition problems. Diarrhea also remains one of the leading causes of mortality in children from low and medium income countries [1].

Clean water and sanitation play an essential role in protecting human health during crisis and disease outbreaks. According to a WHO/UNICEF 2014 report, clean water sources were not available in 58% of Ethiopian rural areas. A National Water, Sanitation, and Hygiene Inventory from 2012 reported that only 32% of health facilities in Ethiopia have access to safe water. In Ethiopia, the children under five had a mortality rate of 59 deaths per 1,000 live births and diarrhea was the third leading cause of mortality in 2015 [2–5].

In this study we characterize the enteric viromes in children under-five years old in the Amhara region of Ethiopia in the context of a cluster-randomized trial of a water improvement intervention for trachoma. Description of these fecal viruses provide a baseline against which future viromes from the same population can be compared to monitor longitudinal changes in the composition and prevalence of circulating viruses.

## Materials and methods

### Study design

The virome analysis described in this report is a non-pre-specified secondary analysis from a cluster-randomized trial of a water improvement intervention for trachoma (clinicaltrials.gov NCT02373657). The primary outcome for the trial was ocular chlamydia. Fourteen communities in rural Ethiopia were selected for the trial, with half randomized to a water point intervention and the other half randomized to no intervention. The intervention consisted of building a new hand dug water well in each community. Stool samples were collected from 0–5 year-old children during the final 24-month study visit of the trial.

### Study population and selection

The cluster-randomized trial study took place in a rural agrarian region in the Goncha Siso Enese district (*woreda)* of Amhara, Ethiopia. *Woredas* in Ethiopia are divided into administrative units known as *kebeles*, and at the time of the study, *kebeles* were subdivided into government-defined units known as state teams. State teams, which consisted of approximately 275 people in our study area, are termed communities for this report.

Communities had been participating in a series of cluster-randomized trials testing different mass drug administration strategies for trachoma elimination since 2006 (clinicaltrials.gov #NCT00322972). As part of these trials, 72 communities had received some form of mass azithromycin distribution for trachoma at least annually from 2010 to 2013. Methods for these trials are described in detail elsewhere [6]. From these 72 communities we randomly selected fourteen that were relatively accessible (<1 hour walk from the farthest place a four-wheel drive vehicle could reach) and had poor access to water (only one or no water well). The baseline visit for the trial occurred in April 2014 and the final study visit occurred in April 2016. April is the dry season in this region.

A door-to-door population census was taken in all communities before the study visit. All children aged 0–5 years (i.e., up to but not including the sixth birthday) enumerated on the census were eligible to participate in the study.

### Stool sample collection

Caregivers were instructed to have their child defecate in a plastic child’s potty chair lined with a black plastic bag. For children unable to produce a stool within two hours, supplies were provided to the caregiver, with instructions to collect stool at home the following morning, and bring it to a collection site the following day at a designated time.

At the time the stool sample was returned, 0.5ml of stool was placed in a 1ml plastic tube. The sample was immediately put on ice and transferred to a -20 Celsius freezer at the end of the day. At the completion of the sample collection, in early May 2016, all samples were transferred to Bahir Dar Regional Laboratory (Bahir Dar, Ethiopia) and kept at -20 Celsius until they were shipped to University of California, San Francisco in February 2017.

### Viral metagenomics

Approximately 0.1 gram of fecal matter from 269 stool samples were assembled into 29 pools of six to twelve samples either from villages with or without water improvement. To reduce possible batch effects, pools from the control and the intervention groups were processed in an inter-digitated manner. Pools were first clarified by 15,000g centrifugation for ten minutes, and supernatants filtered using a 0.45-μm filter (Millipore). Nucleic acids in the filtrates were digested with a mixture of nuclease enzymes and viral nucleic acids were then extracted using a Maxwell 16 automated extractor (Promega) [7]. Random RT-PCR followed by Nextera™ XT Sample Preparation Kit (Illumina) were used to generate a library for Illumina MiSeq (2 × 250 bases) with dual barcoding as previously described [8, 9].

### Bioinformatic analyses

#### Overview

An in-house analysis pipeline was used to analyze sequence data. Raw data was first pre-processed by subtracting human and bacterial sequences, duplicate sequences, and low quality reads. The reads were de novo assembled and contigs and singlet reads were aligned against a customized viral proteome database using BLASTx. Candidate viral hits were then compared to a non-virus non-redundant (nr) protein database to remove false positive viral hits.

#### Database compilation

To electronically subtract non-viral sequences the human reference genome sequence (hg38) and mRNA sequences were first concatenated. Bacterial nucleotide sequences were also extracted from NCBI nt fasta file [10] based on NCBI taxonomy [11]. Human and bacterial nucleotide sequences were then compiled into bowtie2 (version 2.2.4) databases [12] for human and bacterial sequences subtraction. Two databases were constructed: 1) virus BLASTx database was compiled using NCBI virus reference proteome [13] to which was added viral protein sequences from NCBI nr fasta file (based on annotation taxonomy in Virus Kingdom); and 2) a non-virus nr (NVNR) database was compiled using non-viral protein sequences extracted from NCBI nr fasta file (based on annotation taxonomy excluding Virus Kingdom). Repeats and low-complexity regions were masked using segmasker from blast+ suite (version 2.2.7)[14].

#### Preprocessing

Paired-end reads of 250 bp generated by MiSeq were debarcoded using vendor software from Illumina. Human host reads and bacterial reads are identified and removed by mapping the raw reads to human reference genome hg38 and bacterial genomes release 66 using bowtie2 in local search mode with other parameters set as default, requiring finding 60bp aligned segments with at most 2 mismatches and no gaps [12]. Reads were considered duplicates if 5bp to 55bp from 5’ end are identical. One random copy of duplicates was kept. Duplicate sequences were replaced with sequence ‘A’ as a place holder; preserving the original order of the paired-end files for paired-end sequence assembly. A paired-end sequence record is removed if both paired reads are deleted duplicates. Low sequencing quality tails were trimmed using Phred quality score 20 as the threshold. Adaptor and primer sequences were trimmed using the default parameters of VecScreen using default parameters [14].

#### De novo assembly

We developed a strategy that integrates the sequential use of various de Bruijn graph (DBG) and overlap-layout-consensus assemblers (OLC) with a novel partitioned sub-assembly approach called ENSEMBLE [15].

Both single reads (singlets) and de novo assembled contiguously overlapping reads (contigs) were first analyzed using BLASTx (version 2.2.7) for translated protein sequence similarity to all viral protein sequences in GenBank’s virus RefSeq database plus protein sequences taxonomically annotated as viral in GenBank’s non-redundant database. An initially non-stringent E-value cutoff of <0.01 was selected in order to identify even weakly matching potential viral sequences. To remove background due to sequence misclassification these initial viral hits were then compared to all protein sequences in NR using the program DIAMOND (version 0.9.6) and retained only when the top hit was to a sequence annotated as viral. A threshold E score of <10^−10^ was then used to ensure only reads with high levels of similarity to viral proteins were counted. Further analyses focused on eukaryotic viruses.

To align singlets and contigs to reference viral genomes from GenBank and generate complete or partial genome sequences the Geneious R10 program was used. For plotting read numbers to different viral clades the number of reads with BLASTx E score <10^−10^ to named viruses was divided by the total number of reads multiplied by 10^4^ then log 10 transformed to determine the size of the colored circles using Excel.

### Phylogenetic analyses

Phylogenetic trees were constructed from VP1 amino acid sequence for picornaviruses or nucleotide for norovirus RdRp region. Evolutionary analyses were conducted in MEGA6 using the Neighbor-Joining method [16]. Percentage bootstrap values from 1000 replicate trees are shown [17]. All positions with less than 95% site coverage were eliminated.

### Statistical methods

All statistical analyses were performed in R version 3.4.2 (R Foundation for Statistical Computing, Vienna, Austria) using R Studio version 1.1.383. The number of virus matching singlets (E score <10^−10^) for each sample pool along with their viral taxonomic assignments and sample characteristics were analyzed using the ‘phyloseq’ package [18]. The ‘phyloseq’ package was used to calculate alpha diversity measures, which were then plotted using boxplots in ‘ggplot’[19]. A Kruskal-Wallis test was then used to evaluate if differences in alpha diversity measures were statistically significant between the control and intervention groups.

### Data availability

The genomes of viruses are available on the NCBI website; the accession numbers are given in Tables 1 and S1. The raw sequence data is available at NCBI’s Short Reads Archive under GenBank accession number SRP120619.

**Table 1:** Characteristics of mammalian viral contigs.

Family	*Genus*	Species	Genotypes			Pool ID #	GenBank accession number	Length of genome assembled (% sequenced)	Reference genome GenBank accession number	Region of reference genome covered	Nucleotide similarity with reference	aa idendity to VP1
***Picornaviridae***	Enterovirus	Enterovirus A	Coxsackievirus A6			P11	MG692404	3597 (100%)	KX064297	3712_7309	84.7%	
						P20	MG692405	3729 (61.1%)	KX064297	922_7023	82.1%	
						P22	MG692406	2782 (76.6%)	KX064297	4216_7278	84.8%	
			Coxsackievirus A14			P25	MG692407	5425 (77.7%)	KP036482	197_7176	82.6%	
			Coxsackievirus A16			P4	MG692408	2203 (82.8%)	JQ746670	1950_4673	85.0%	
						P11	MF990299	2607 (100%)	JQ746670	1068_3674	85.9%	99.3%
						P12	MF990300	7225 (100%)	JQ746670	101_7325	82.6%	99.3%
		Enterovirus B	*Echovirus E6*			P17	MG692409	4713 (77.5%)	KT353725	23_6013	85.4%	
						P22	MG692410	2315 (73.9)	HM852755	3631_6762	83.2%	
			*Echovirus E14*			P14	MF990302	6462 (100%)	AY302540	1_6462	79.8%	93.6%
						P19	MF990305	7333 (99.2%)	AY302540	1_6505	79.9%	93.6%
			*Echovirus E16*			P4	MF990293	7392 (100%)	AY302542	24_7421	80.6%	97.2%
						P7	MG525060-62	891 (49.4%)	KP289436	1131_2933	80.4%	
			Echovirus E18			P12	MF990301	7270 (100%)	KX139457	91_7362	81.3%	94.8%
						P18	MG692411	1642 (64.2%)	KX139456	1317_3871	80.3%	
						P26	MG692412	2982 (87.7%)	KX139456	1311_4709	80.7%	
			*Echovirus E19*			P3	MF990292	7274 (100%)	AY302544	70_7344	79.4%	92.4%
						P14	MF990303	3988 (99.2%)	AY302544	1025_5053	79.4%	92.1%
			*Echovirus E27*			P8	MF990295	7167 (100%)	AY302551	207_7376	78.8%	89.2%
		Enterovirus C	*Coxsackievirus A1*			P8	MF990294	7160 (100%)	AF499635	197_7357	83.2%	89.8%
			*Coxsackievirus A13*			P21	MG692413	3489 (65.4%)	JF260922	1496_6832	79.3%	
			*Coxsackievirus A17*			P9	MF990296	6215 (100%)	AF499639	661_6875	80.9%	95.0%
						P18	MG692414	4076 (81.1%)	AF499639	1216_6240	79.4%	
						P26	MF990306	3147 (58.3%)	AF499639	1774_7164	80.9%	93.4%
						P28	MF990307	6525 (100%)	AF499639	748_7272	81.9%	95.0%
			*Coxsackievirus A20*			P9	MF990297	6082(100%)	DQ358078	803_6885	83.8%	97.6%
						P10	MF990298	6541 (100%)	DQ358078	311_6852	83.3%	98.3%
						P15	MG692415	4040 (66.1%)	DQ358078	732_6839	83.5%	
						P16	MF990304	7280 (99.2%)	DQ358078	55_7392	84.5%	98.0%
			*Enterovirus C99*			P20	MF990308	1950 (96.2%)	EF015009	1296_3320	81.6%	94%*
						P13	MG560270	4280 (68.9)	EF015009	852_7061	82.4%	
	*Cosavirus*	*Cosavirus A*	*cosavirus A_12*			P4	MF621606	3344 (68.3%)	JN867774	1_690	90.0%	96.8%*
			* *			P8	MG692416	1473 (50.4%)	FJ438902	4069_6987	90.9%	
						P10	MG692417	900 (50.5)	FJ438902	5635_7416	88.0%	
			*cosavirus A_8*			P11	MF621609	6047 (88.3%)	JN867776	1_905	85.2%	98.2%*
			*cosavirus A_5*			P12	MF621608	5728 (91.3%)	JN867785	1_694	85.0%	97%*
			* *			P14	MG692418	336 (100%)	FJ438904	1234_1599	86.3%	
						P16	MG525054-56	1179 (38.49%)	FJ438902	4354_7416	89.6%	
						P21	MF621610	1987 (56.4%)	FJ438902	3850_7374	86.9%	
						P25	MG525057-59	1758 (35.8%)	AB920345	1278_6182	89.8%	
		*Cosavirus D*	*Cosavirus D1*			P9	MF621607	5330 (96.8%)	NC012802	672_6173	83.0%	94.5%
		*Cosavirus E/D*	* *			P16.2	MG692419	672 (100%)	JN867757	4699_5370	91.0%	
						P26	MF621611	2501 (81.5%)	JN867757	3436_6501	91.3%	
		*Cosavirus E*	*Cosavirus E*			P2	MF621605	2391 (77.7%)	FJ555055	2770_5844	85.9%	
	*Parechovirus*	*Human parechovirus 1*	* *			P1	MG438289	5070 (74%)	EF051629	254_7096	86.8%	96.9%
						P5	MG026486	7041 (99%)	EF051629	165_7272	85.7%	96.1%
						P6	MG026487	7054 (100%)	EF051629	203_7256	85.4%	96.5%
						P13	MG692434	1597 (68.4%)	EF051629	319_2653	89.2%	
						P16	MG026489	7091 (99.5%)	EF051629	159_7286	83.1%	96.5%
						P21	MG026491	5965 (86.8%)	EF051629	245_7115	86.5%	96.5%
						P28	MG026490	7102 (100%)	EF051629	173_7274	85.7%	96.1%
		*Human parechovirus 4*	* *			P3	MG692433	1078 (96%)	DQ315670	568_1689	88.2%	
		*Human parechovirus 5*	* *			P9	MG026488	6877 (98.8%)	HQ696575	148_7109	81.4%	92.8%
		*Human parechovirus 6*	* *			P20	MG438290	3506 (66.8%)	AB252582	565_5812	94.6%	95.8%*
		*Human parechovirus 8*	* *			P25	MG026492	2622 (91.6%)	EU716175	154_3006	82.8%	97.6%*
		*Human parechovirus 17*	* *			P26	MG438291	6606 (100%)	KT319121	334_6936	81.3%	97.3%
	*Hepatovirus*	*Hepatovirus A*	Hepatovirus A_IB			P5	MF621612	3819 (78.1%)	M20273	1759_6642	93.9%	99.5%
						P11	MF621613	4062(81.4%)	M20273	1819_6807	93.5%	99.5%*
						P16	MF621614	7209(100%)	M20273	159_7368	94.6%	100.0%
						P18	MF621615	5511 (84.4%)	M20273	150_6672	94.7%	100%*
	*Kobuvirus*	* Aichivirus A*	* *			P4	MG009596	7917 (98.2%)	FJ890523	3_8059	96.4%	98.6%*
						P6	MG692430	4213 (57.1%)	FJ890523	411_7780	96.3%	
						P9	MG692431	5632 (72.7%)	FJ890523	226_7966	96.6%	
						P24	MG692432	3322 (63.4%)	FJ890523	407_5644	96.6%	
	*Salivirus*	*Salivirus*	* *			P2	MG026493	6452 (93.1%)	KT240115	968_7895	91.0%	92.3%
						P3	MG692420-21	1476 (49.2%)	KT310068	4519_7512	96.5%	
						P6	MG026494	6866 (100%)	KT310068	955_7820	95.8%	97.1%
						P14	MG026495	7587 (99.7%)	KT310068	225_7827	95.6%	97.1%
						P25	MG692422-24	1082 (23%)	KM023140	1730_6292	91.3%	
						P26	MG692425-28	2034 (45.8%)	KT310068	1087_5520	93.4%	
						P27	MG692429	459 (100%)	KT310068	3043_3501	95.4%	
						P28	MG026496	7440 (95%)	NC_012957	8_7839	91.1%	95.5%*
				*Orf1*	*Orf2*							RdRp region nt similarity
***Caliciviridae***	*Norovirus*	*Norwalk virus*	*Norovirus GI*	*GI*.*3*	*GI*.*3*	P8	MG557648	6257 (85.2%)	KJ196292	272_7613	89.5%	91.8%**
				*GI*.*7*	* *	P10	MG572183	588 (98.5%)	KU311161	4803_5390	86.1%	85.6%**
				*GI*.*7*	*GI*.*7*	12	MG557649	4663 (72.5%)	KU311161	369_6795	92.6%	94.5%**
				*GI*.*3*	*GI*.*3*	P15	MG557650	7425 (100%)	KJ196292	74_7498	89.4%	91.4%**
				*GI*.*7*	*GI*.*7*	P20	MG557651	7012 (95.3%)	KU311161	1_7351	91.1%	90%**
				*GI*.*6*	*GI*.*6*	P26	MG557652	4702 (66%)	AF093797	392_7498	91.1%	91%**
			*Norovirus GII*	*GII*.*7*	*GII*.*6*	P3	MG557654	7236 (100%)	KU935739	179_7414	97.8%	98.9%
				*GII*.*e*	*GII*.*10*	P4	MG557655	6351 (100%)	JX459907	236_6595	86.5%	95.5%
				*GII*.*7*	*GII*.*9*	P6	MG557656	2999 (58%)	AB039777	68_5180	89.2%	91.8%**
				* *	* *	P8	MG557653	3388 (79.5%)	EF187497	422_4681	82.0%	
				*GII*.*e*	*GII*.*4*	P11	MG557657	4508 (64.4%)	JX459907	356_7345	95.3%	96%**
	*Sapovirus*	*Sapporo_virus*	*Sapporo_virus*			P5	MG692435	3804 (58.4%)	AJ249939	350_6856	94.8%	
						P19	MG692436	3898 (54.4%)	AJ249939	152_7311	95.0%	
						24	MG692437	3162 (55.9%)	AY237420	686_6337	94.9%	
** **												aa identity to NS1
***Parvoviridae***	*Bocaparvovirus*	*Primate_bocaparvovirus_1*	*Human_bocavirus_1*			P20	MG383449	5155 (100%)	KX373884	121_5275	99.4%	99.7%
			*Human_bocavirus_3*			P2	MG383445	4195 (87.8%)	FJ973562	133_4912	95.6%	98.5%
						P27	MG522845-6	1065 (40%)	KM624026.1	2354_5003	97.3%	
		*Primate__bocaparvovirus_2*	*Human_bocavirus_2*			P4	MG383447	5204 (100%)	EU082213	1_5204	98.8%	99.8%
						P9	MG522843	562 (100%)	EU082213	2081_2642	98.8%	
						P12	MG522844	652 (100%)	EU082213	2066_2717	98.6%	
						P15	MG383448	3401 (72.1%)	EU082213	434_5149	96.5%	
						P25	MG383450	5155 (100%)	FJ170279	1_5172	98.4%	100.0%
			*Human_bocavirus_4*			P3	MG383446	5269 (100%)	KC461233	49_5207	99.3%	99.8%
						P29	MG522847	2538 (66.4%)	KC461233	480_4299	99.2%	
	*None*	*Bufavirus-3*	*Bufavirus-3*			P4	MG550916	321 (100%)	AB982221	3895_4215	97.0%	
						P16	MG550917	183 (100%)	AB982221	2416_2598	96.7%	
***Picobirnaviridae***	*Picobirnavirus*	* *	*Picobirnavirus*			P6	MG522848	447 (87%)	KJ206568.1	724_1236	91.0%	
						P25	MG522849	474 (100%)	AF246939.1	667_1140	91.0%	

*3–29% gaps in VP1 protein alignments,

**1 to 67% gaps in RdRp region nucleotide alignments

### Ethics statement

Ethical committees at the University of California (San Francisco, CA, USA); Emory University (Atlanta, GA, USA); The Food, Medicine and Health Care Administration and Control Authority of Ethiopia; and the Ethiopian Ministry of Science and Technology granted approval for this study. We obtained verbal informed consent in Amharic from the parent or guardian of each study participant.

## Results

### Characteristics of study population

**Fig 1:**
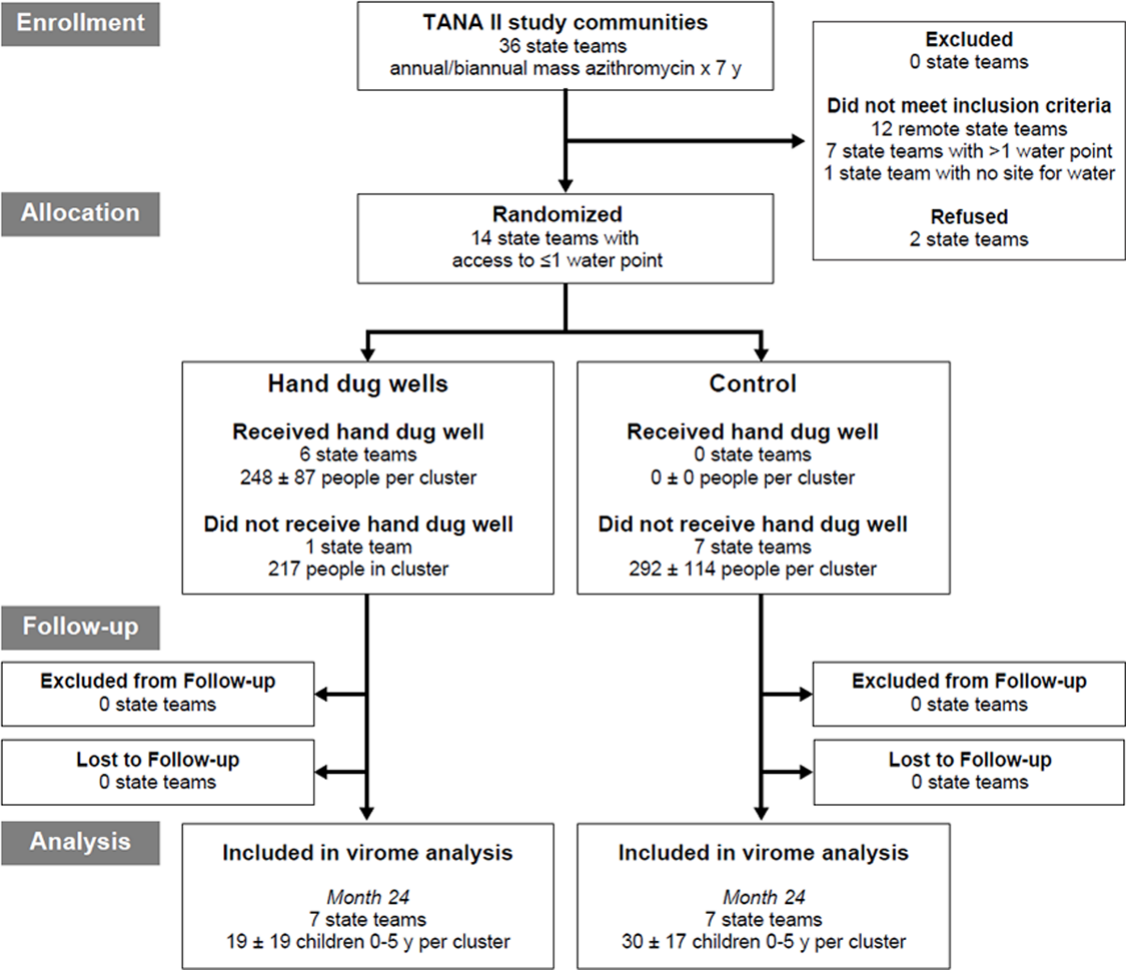
Flow diagram for collection of fecal samples.

A flow diagram of sampling and participation is shown (Fig 1). Of 446 censored children who were eligible to participate, 317 children presented for the study visit examination and 269 provided stool samples. The mean age of children with stool samples was 2.7 years old, 56.5% (152/269) of children were female.

Pools of fecal samples were then processed by filtration and nuclease treatment to digest non-capsid protected nucleic acids. Viral genomes where then extracted and DNA and RNA randomly amplified and sequenced on the Illumina MiSeq platform (250 bases paired end reads). A total number of 27.8 million reads were generated for an average number of reads of approximately one million per pool. The raw sequence data for each pool is available at NCBI’s Short Reads Archive under GenBank accession number **SRP120619**.

**Fig 2:**
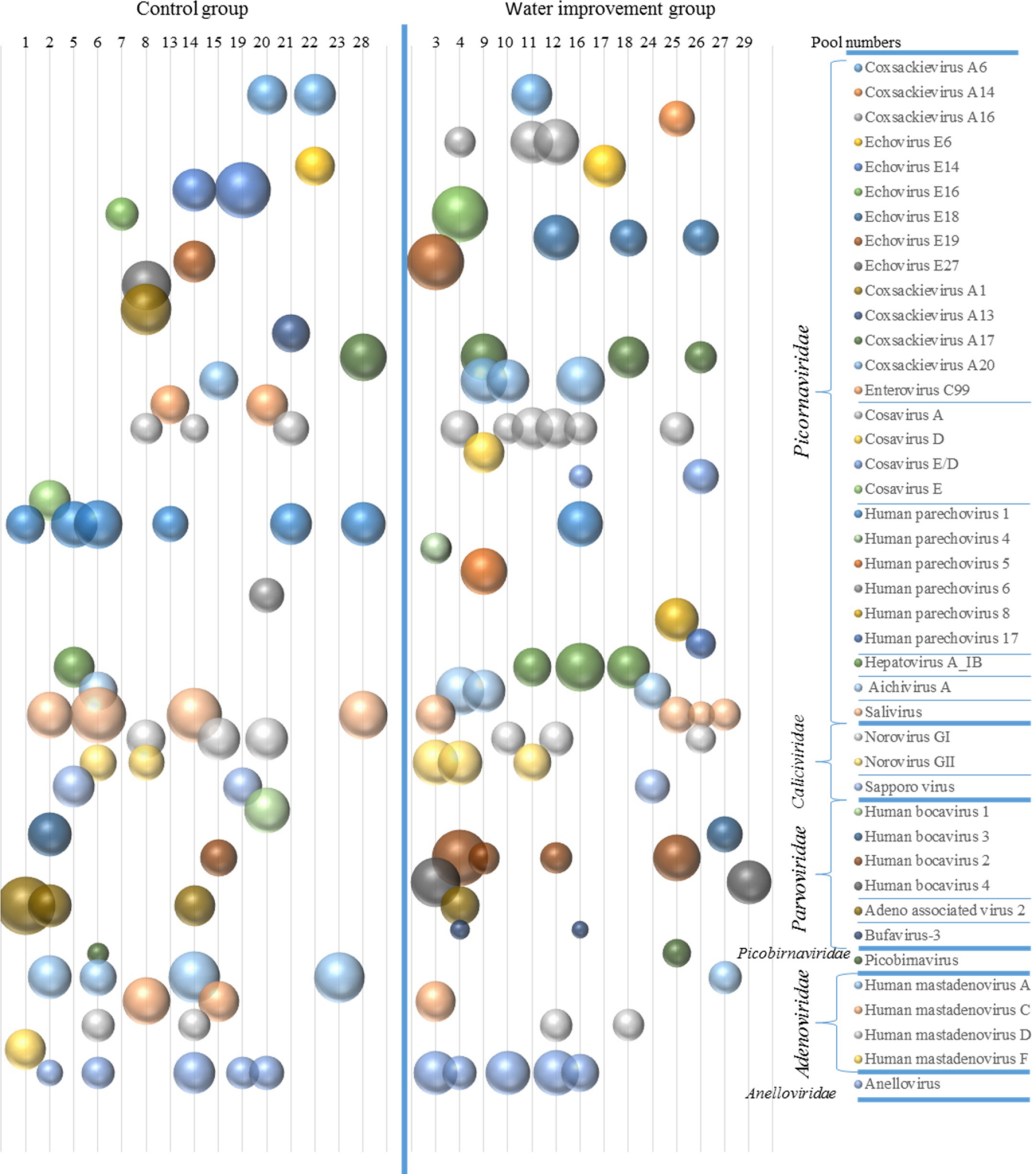
Distribution of viral sequences reads to named viruses using BLASTx E score <10^−10^.

The most commonly detected viral reads belonged to the *Picornaviridae* family which were detected in 27/29 (93.1%) pools. 0.90% (249,982) of 27.8 million total sequence reads, were found to encode *Picornaviridae* related proteins (E scores <10^−10^). The fraction of the 29 sample pools analyzed that were positive for members of six different *Picornaviridae* genera were: Enterovirus (72.4%), Parechovirus (41.3%), Cosavirus (41.3%), Salivirus (27.5%), Kobuvirus (13.7%), and Hepatovirus (13.7%). Next in prevalence, *Caliciviridae* family members were detected in 44.8% of pools and consisted of norovirus GI (20.6%), norovirus GII (17.2%) and sapporovirus (10.3%). *Parvoviridae* family members were also detected in 41.3% of the pools including primate bocaparvovirus 1 and 2 (34.4%), adeno-associated virus 2 (13.7%), and bufavirus 3 (6.8%). In the *Adenoviridae* family human_mastadenoviruses A species (HAdV-A) was detected in 17.2% of pools, HAdV-C in 10.3%, HAdV-D in 13.7%, and HAdV-F in 3.4%. Picobirnavirus sequences were found in 2/29 (6.8%) of the pools. No rotavirus nor astrovirus sequence reads were detected. The fraction of total reads from each pool encoding proteins with high-level similarity (E scores <10^−10^) to different human viruses is shown (Fig 2).

For the viruses that yielded the largest number of reads complete or more partial genome sequences were separately assembled from each of the 29 libraries. Nucleotide sequence reads from each library were aligned against the GenBank available genomes that showed the greatest translated protein similarity. Single large contigs of nearly complete genomes, or multiple contigs aligned to the same reference genome but with gaps remaining between mapped segments, were generated (Table 1). These assembled viral sequences were then compared to taxonomically classified genomes. The results are presented as % amino acid identity for proteins used for genotype classification (VP1 of picornaviruses) or when not available as % nucleotide identity determined using BLASTn (Table 1).

### Family *Picornaviridae*: Enteroviruses

**Fig 3:**
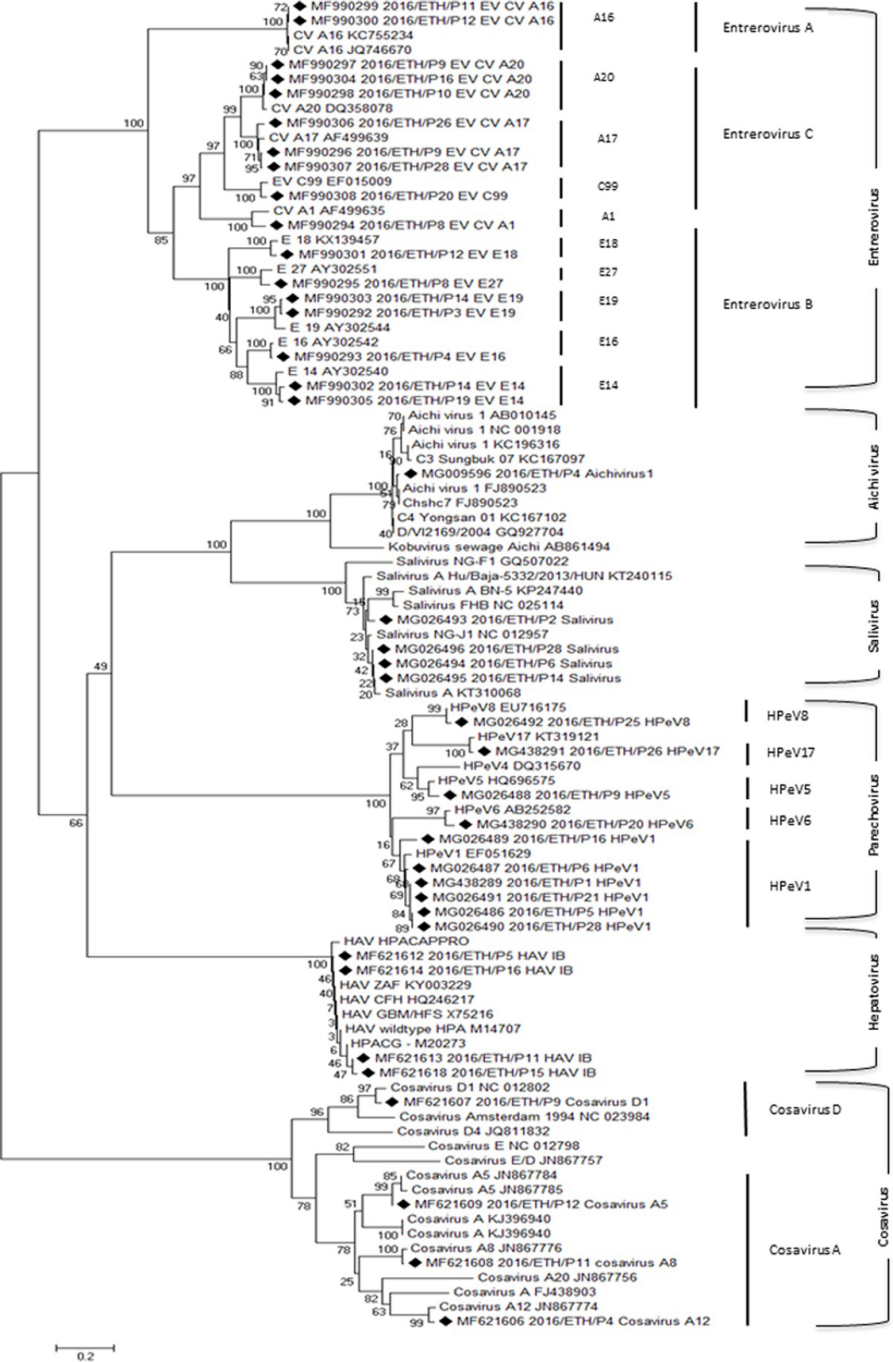
Phylogenetic analysis of VP1s from different genera of the *Picornaviridae* family. Viral sequences described here are highlighted by black diamonds.

Thirty one near complete or partial enterovirus genomes ranging in size from 891 nucleotides (nt) to 7,392 nt were generated, 17 of which included the VP1 capsid region. A phylogenetic analysis of the VP1 of enteroviruses and other *Picornaviridae* genera is shown (Fig 3).

#### Enterovirus species A

Seven enterovirus A infections were identified. Two enterovirus A (EV-A) Coxsackievirus A16 (CV_A16) sequences from different pools showed 99.3% VP1 region amino acid closest identity to CV-A16 genomes in GenBank. Five other EV-A sequences without VP1 capsid region showed 82.1 to 85% nucleotide closest identity to three different enterovirus species A genotypes yielding three genotypes Coxsackievirus A6, one Coxsackievirus A14, and another Coxsackievirus A16 partial genomes. The two CV_A16 with VP1 showed 0 amino acid substitution per site and their available genome sequences (Table 1) shared 99.3% overall similarity indicating a recent common origin.

#### Enterovirus species B

Twelve enterovirus B infections were identified. Seven enterovirus B (EV-B) contigs containing the VP1 capsid region were generated. These sequences showed 89.2 to 97.2% VP1 region amino acid closest identity to five different enterovirus B genotypes (two Echovirus E14, one Echovirus E16, one Echovirus E18, two Echovirus E19, and one Echovirus E27) reported in GenBank (Table 1). The genotypes detected twice (echovirus E14 and E19) with complete polyprotein coding genome regions showed 0.025 and 0.006 amino acid substitutions per site respectively. Pair-wise alignment showed nucleotide identity of 90.5 and 94.0% similarities respectively. Five EV-B sequence contigs without VP1 capsid region showed 82 to 85.4% nucleotide identity to three enterovirus B genotypes (two echovirus E6, one echovirus E16, and two echovirus E18) reported in GenBank (Table 1).

#### Enterovirus species C

Twelve enterovirus C infections were identified. Four different genotypes of enterovirus C (EV-C) were detected showing 89 to 98.3% VP1 region amino acid identity to reference enterovirus C genotypes. One Coxsackievirus CV-A1, one EV-C99, three Coxsackievirus CV-A17, and three Coxsackievirus CV-A20 viruses could be identified. The complete VP1 coding sequences of the twice detected CV-A17 (excluding the more divergent CV-A17 from pool 9) and the thrice detected CV-A20 showed 0.012 and 0.0–0.012 amino acid substitutions per site respectively. Pair-wise alignment showed nucleotide identity of 98.0 and 94.6–98.3% similarities respectively again reflecting a recent common origin. Four other EV-C sequence contigs without VP1 capsid region showed 79 to 85% nucleotide identity to enterovirus C genotypes (coxsackievirus A13, coxsackievirus A17, coxsackievirus A20, enterovirus C99) reported in GenBank (Table 1).

### Family *Picornaviridae*: Parechoviruses

Twelve human parechovirus infections were detected, 10 of which generated complete VP1 sequences. Six VP1 showed closest amino acid identity (96.1 to 96.9%) to human parechovirus 1 (HPeV1). One HPeV5, one HPeV6, one HPeV8, and one HPeV17 viral sequences were also detected showing closest amino acid identity of 92.8, 95.8, 97.6 and 97.3% respectively to their respective genotype VP1. The two non-VP1 contigs showed 89.2 and 88.2% nucleotide identity to HPeV1 and HPeV4. Two pairs of very closely related HPeV-1 VP1 sequences showed 0.006–0.008 amino acid substitutions per site. When their contigs were compared they showed nucleotide similarities of 98.3 and 98.5% indicating a recent common origin for both pairs.

### Family *Picornaviridae*: Hepatoviruses

Four hepatovirus A infections were detected. Four of the observed contigs included the VP1 region and showed closest amino acid identity from 99.5 to 100% to hepatovirus A genotype IB genome available in GenBank. When the four contigs were aligned, their overlapping regions showed nucleotide identity of 95.2–99.9%. Two pairs of very closely related hepatovirus A VP1 sequences showed 0.006 and 0.008 amino acid substitution per site, respectively. When their contigs were compared they showed nucleotide similarities of 95.4 and 99.7%, respectively indicating a recent common origin for both pairs.

### Family *Picornaviridae*: Saliviruses

Eight salivirus infections were detected, 4 of which included the VP1 capsid region. Three sequences showed 92.3 to 97.1% VP1 amino acid identity to Salivirus_A strain GUT/2009/A-1746 from Guatemala, while the fourth VP1 was closest (95.5%) to Salivirus_NG-J1 from Nigeria. These four contigs of nearly complete coding sequences showed 87.3 and 98% nucleotide identity over at least 6452 bp. Four other contigs showed 91.3 to 96.5% nucleotide identity to other salivirus strains reported in GenBank. Three saliviruses with very closely related VP1 sequences (excluding the more divergent pool 2 salivirus) showed 0–0.06 amino acid substitutions per site. These 3 contigs showed nucleotide similarities of 97.8–99.3% similarity, again indicating a recent common origin for these 3 viruses.

### Family *Picornaviridae*: Kobuviruses

Four kobuvirus infections were detected, only 1 of which included the VP1 capsid region. This VP1 showed 98.6% region amino acid identity to Aichi virus 1 isolate Chshc7 from China. The three other viral sequences showed nucleotide identity of 96.3 to 96.6% to other Aichi viruses 1.

### Family *Picornaviridae*: Cosaviruses

Thirteen cosavirus infections were detected. Four of these sequences included the VP1 region and showed closest amino acid identities of 97, 98.2, 96.8 and 94.5%, respectively, to an HCoSV_A5 genotype, HCoSV_A8 genotype, HCoSV_A12 genotype, and HCoSV_D1 genotype. Nine cosavirus sequences without VP1 capsid region showed 85.9 to 91.9% nucleotide identity to Cosavirus A (six sequences), cosavirus E (one sequence) and cosavirus E/D (two sequences) reported in GenBank. In total, 9 HCoSV_A (species A), 1 HCoSV_D, 2 HCoSV_E/D, and 1 HCoSV_E viral sequences, were identified and the near complete or partial genomes submitted to GenBank.

### Family *Caliciviridae*

**Fig 4:**
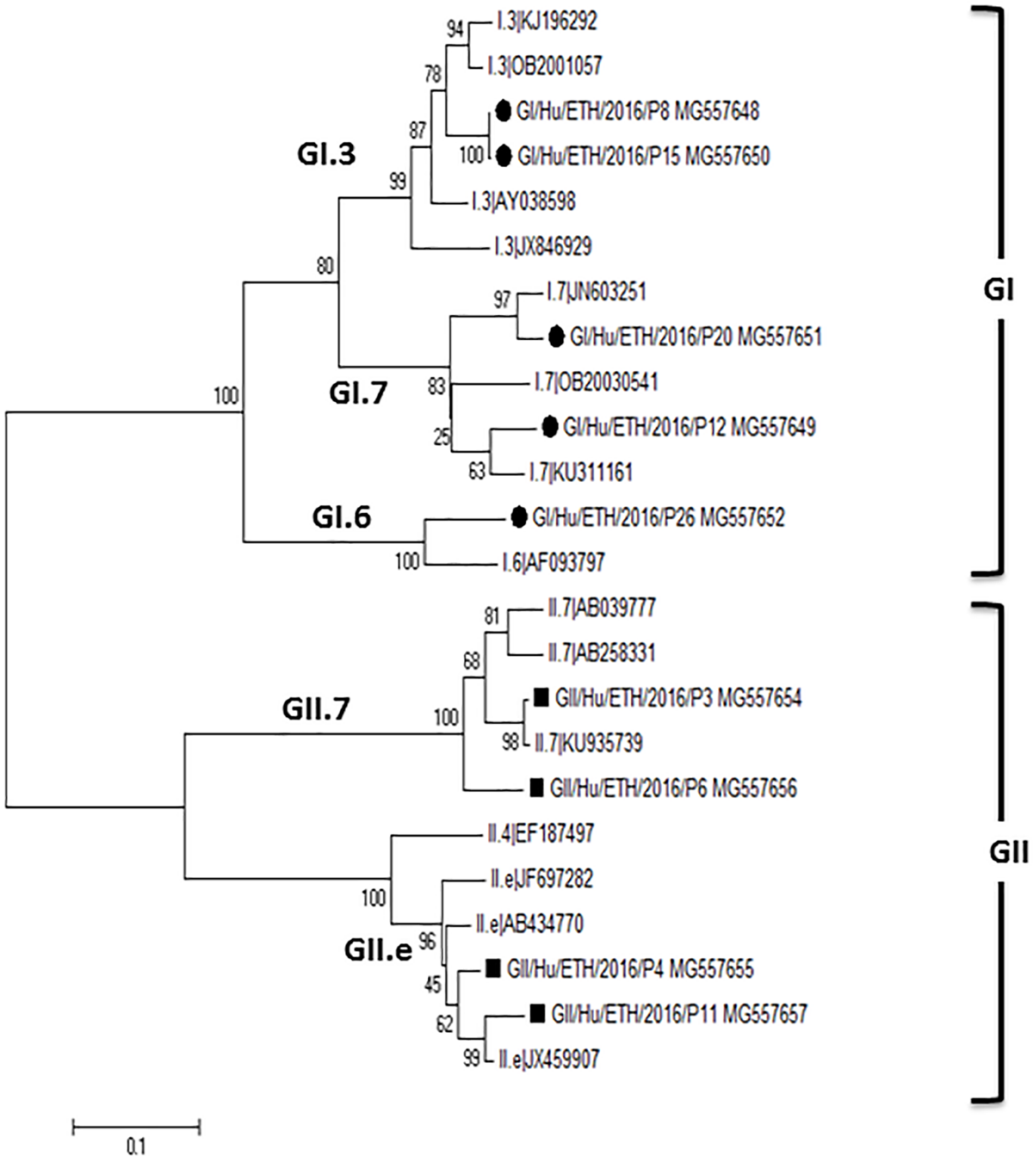
Phylogenetic analysis of RdRp from different genotypes of noroviruses. Viral sequences described here are highlighted by black diamonds.

Eleven noroviruses viral infections were detected, 10 of which included the regions used for genogroup determination (partial RdRp) and 9 also included ORF2 for capsid genotyping. To determine genogroups and capsid genotypes the Norovirus Genotyping Tool was used [20]. 5 genogroup I (two GI.P3, two GI.P7, and one GI.P6) and 4 genogroup II (two GII.Pe and two GII.P7) were identified. The ORF2 genotyping results were identical for GI but for GII viruses genotypes GII.6, GII.10, GII.9, and GII.4_Sydney_2012 capsid were reported. A phylogenetic analysis of the partial RdRp region of these noroviruses is shown (Fig 4).

Three Sapporo virus sequences were also found which showed 94.8–95% nucleotide identity to SLV/Bristol/98/UK and Sapovirus Mc10. The overlapping region of the 3 contigs showed nucleotide identities of 72 to 99.5%.

### Family *Parvoviridae*: Bocaparvovirus

A total of ten bocavirus infections were detected. Five bocavirus NS1 contigs were generated which showed closest amino acid identity of 99.7% to HBoV_1, two showed closest amino acid identity of 99.8–100% to HBoV_2 genome, one showed closest amino acid identity of 98.5% to an HBoV_3 genome, and one showed closest amino acid identity of 99.8% to HBoV_4. Five non-NS1 containing contigs, three showed 96.5–98.8%, one showed 97.3%, and one showed 99.2% nucleotide identity to HBoV2, HBoV3 and HBoV4 respectively. All together, we detected one bocavirus 1, five bocavirus 2, and two bocavirus 3 and two bocavirus 4.

### Family *Parvoviridae*: Dependoparvovirus

Four contigs of adeno-associated virus_2 in the dependoparvovirus genus ranging in size from 2730 nt to 4377 nt were identified. Their overlapping region showed a nucleotide similarity of 96.9 to 99.6%.

### Family *Parvoviridae*: Protoparvovirus

Two short contigs of bufavirus 3 in two pools were also identified with 96.7–97% nucleotide identity to bufavirus-3 in GenBank.

### Families *Adenoviridae*, *Anelloviridae*, *Picobirnaviridae*

Sequences from human_mastadenoviruses A species (HAdV-A), HAdV-C, HAdV-D, and HAdV-F in the *Adenoviridae* family ranging in size from 250 nt to 6282 nt, from 1068 nt to 6829 nt, from 250nt to 980 nt, and of 1153 nt were identified in five, three, four, and one pool, respectively.

Two human picobirnavirus contigs, of 474 nt and 513 nt were also generated which both showed 91% nucleotide identity with human picobirnavirus strain 1-CHN-97 and human picobirnavirus VS6600008 respectively.

### Viral families of unknown host tropism

Also generated were nearly complete genomes of ss+RNA posaviruses and husaviruses, both members of the order *Picornavirales*. Contigs related to the *Smacoviridae* family and related genome named hudisaviruses both members of the highly diverse group known as CRESS-DNA viruses (Circular Rep-encoding ss DNA genomes) were also detected (S1 Table). These viruses have been described in human fecal samples but since their cellular host tropisms remain unknown they have not been included in the subsequent virome comparison analysis.

### Virome comparison in control and intervention groups

**Fig 5:**
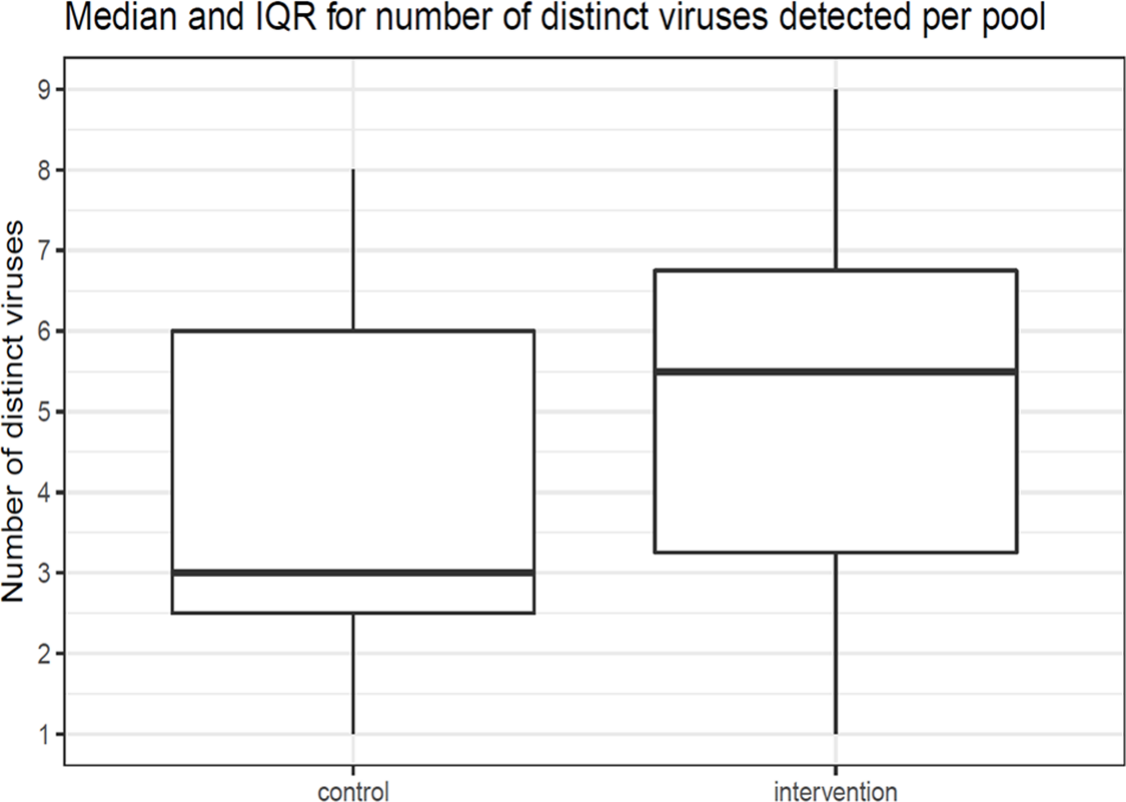
Median and IQR for number of distinct viruses detected per pool of the intervention and control groups.

**Fig 6:**
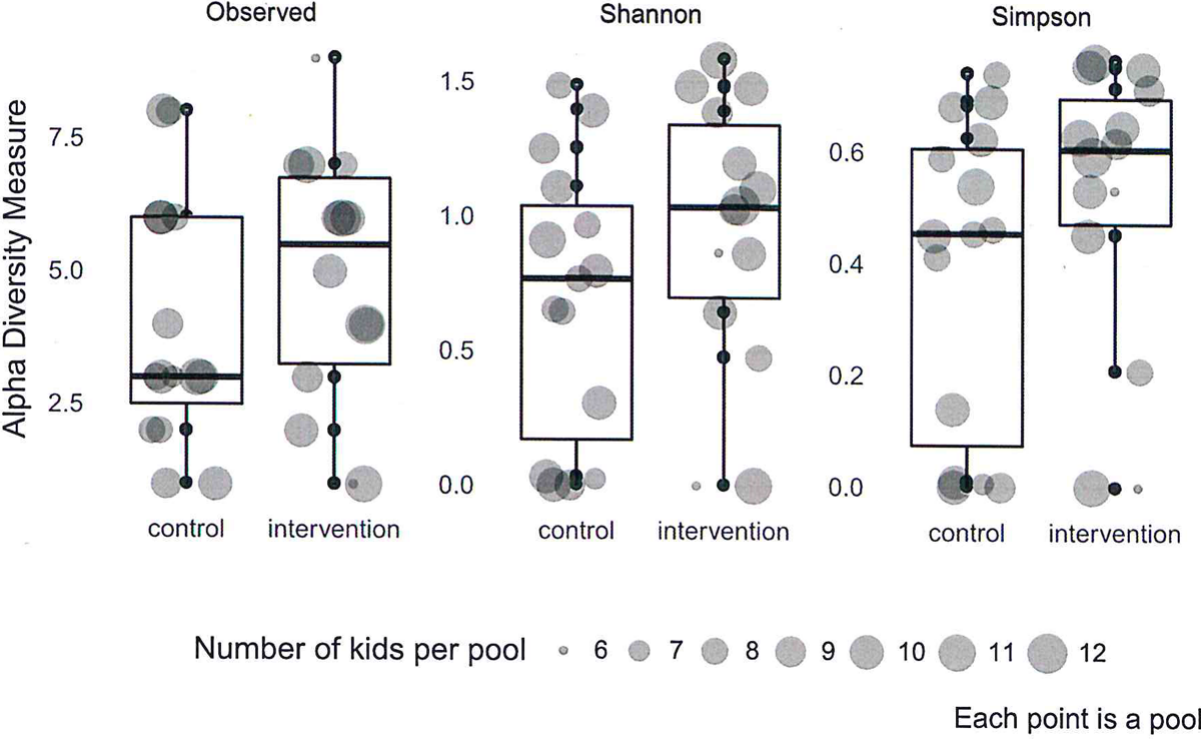
Differences in alpha diversity for the enteric virome between intervention and control groups.

The median number of different human viruses present per pool was 5.5 (IQR 3.25–6.75) in the intervention arm and 3.0 (IQR 2.5–6.0) in the control arm (Fig 5). There was no visual signal for a difference in alpha diversity of the human enteric virome between the intervention and control arm (Fig 6). For each of the three evaluated distance metrics, p-values from the Kruskal-Wallis test evaluating the differences in alpha diversity by intervention arm were non-significant: Richness (observed), p = 0.2893; Shannon, p = 0.2559; and Simpson, p = 0.162.

## Discussion

The high diversity of enteric viruses described in 269 children from 14 Ethiopian villages represents the first description of the enteric virome of East African children. Prior studies in that region have relied on the use of PCR or antigen detection targeting restricted subsets of enteric viruses [21–24].

The fecal samples analyzed were collected as part of a cluster-randomized trial of a water-improvement intervention. Children participating in this trial were randomly sampled from a population census and thus the viromes characterized here are broadly representative for children <5 years old from the Goncha region of Northern Ethiopia in 2016. Availability of this data set can therefore be considered a baseline against which future viromes in that population can be compared to identify sequence changes in the most common viruses and help identify newly introduced or emerging viruses.

The great majority of sequence reads here mapped to RNA viruses of the *Picornaviridae* and *Caliciviridae* families. Picornaviruses showed a particularly high level of genetic diversity including multiple genera, species, and genotypes particularly in the enterovirus, cosavirus, and parechovirus genera. Some picornaviruses had nearly identical VP1 and very closely related genomes (>95%). This high level of similarity between variants from different children reflects recent common origins and point towards those genotypes that, due to either immune, viral, or environmental factors may be spreading particularly efficiently.

Beside picornaviruses, other RNA (caliciviruses, picobirnaviruses) and DNA (adenoviruses, parvoviruses, and anelloviruses) viruses were also detected. Rotavirus sequences were not detected. Globally rotavirus remains a leading cause of severe acute water diarrhea but has shown a significant decline in vaccine age-eligible children in Africa following introduction of rotavirus vaccination [25, 26]. Ethiopia initiated a vaccination campaign in 2013 with an estimated coverage of 85% by 2015 [26], We did not detect any rotavirus in the sample, which may be an indication of successful recent vaccination campaigns or because this was a population-based sample and may not have captured children ill with rotavirus infections. Astroviruses are also common enteric childhood enteric infections [27–30] but none was detected among the population sampled.

Metagenomic studies limited to DNA viruses of feces from 65 rural Kenyan adults with and without HIV infections showed a more restricted virome consisting of adenovirus D, anelloviruses, and papillomaviruses (the last in a single sample)[31]. Reads belonging to the *Circoviridae* family (members of the CRESS-DNA group) were also reported but circoviruses have not been shown to replicate in humans and therefore may represent genomes related to other CRESS-DNA viruses such as the smacoviruses described above. A greater fraction of adenovirus reads could be measured in AIDS patients with CD4 counts <200. The greater number of viral families detected in the current study may be due to greater susceptibility or exposure of children versus adults, socio-economic or geographic difference, and/or the unbiased amplification methods used which targeted only DNA viruses. While we also found adenovirus and anellovirus sequences numerous genera from the DNA *Parvoviridae* family were also detected here. A metagenomics fecal virome study of Malawian twin infants with severe acute malnutrition was also restricted to DNA viruses [32]. The human viruses reported were the ubiquitous anelloviruses, parvoviruses (bocaviruses and dependoviruses), as well as very low levels of papillomavirus and polyomavirus [32].

Viral genomes of unknown cellular origins were detected namely ssRNA+ posaviruses and husaviruses and circular ssDNA smacoviruses and hudisaviruses, all previously reported in human feces. Based on sequence similarity to cDNA from the long worm of pig (Ascaris suum), posaviruses from feces of pigs [33–37] and other mammals [38] have been hypothesized to infect nematodes present in their intestinal track [33]. This possibility was reinforced by the recent description of a similar genome (Hubei picorna-like virus 11) (YP_009336580) showing 80% protein identity to a posavirus sequenced here from a large pig roundworm from China [39]. The detection of posaviruses may therefore reflect the presence of enteric nematodes in Ethiopian children, a frequent occurrence in that country [40]. Husaviruses are distantly related to posaviruses with a similar RNA genome organization and also phylogenetically located in the *Picornavirales* order [41]. Husaviruses were originally detected in feces from men in Amsterdam (HIV positive and negative) and more recently in Vietnamese human and pig feces (BAV31552.1) [38]. While their cellular host(s) are also unknown these related member of the *Picornavirales* order, which also includes fisaviruses from fish gut content [42], rasavirus from rat feces [38], and basavirus from bat feces [38], share a nucleotide composition which groups them with members of that viral order known to infect arthropods [38]. Nematodes and arthropods, both with exoskeleton principally made of chitin, are phylogenetically related and both members of the Ecdysozoa superphylum.

Smacoviruses and hudisaviruses make up two subgroups of the highly diverse CRESS-DNA viruses whose known cellular hosts range from mammals (Circoviridae) and plants (Geminiviridae) to fungi (SsHADV)[43]. Originally described in feces of chimpanzees [44], smacovirus genomes have also been reported in feces from other non-human primates and humans [45], pigs [46–48] other mammals [49–51] and a bird [52]. Hudisavirus DNA has also been reported in human and macaque feces [53, 54]. As for the large majority of the recently described CRESS-DNA genomes the cellular tropism of the smacoviruses and hudisaviruses genomes detected here remains unknown and could consist of human intestinal epithelial cells, parasites in the gut, or originate from viruses in consumed food products.

The viruses detected here represent minimum values for these children’s viromes. It is possible that some viral nucleic acids may have gone undetected due to viral loads being below detection levels. The same library making method and sequencing depth was used for both intervention and control fecal samples that were processed in an interdigitated manner. Limitations of the metagenomics approach used here should therefore equally impact results from both groups.

The human enteric viruses genetically characterized here are transmitted by fecal-oral transmission and also for adenoviruses by the respiratory route. Because enteric viral infections and fecal shedding are typically acute events of limited duration it is unlikely that the viral nucleic acids detected in our 2016 sampling originate from chronic infections initiated prior to the start of the clean water intervention in 2014.

While we did not detect a difference between the prevalence of different virus families nor the median count of viruses across the control and intervention groups of the water improvement trial, we are wary to conclude that the intervention had no effect on the enteric virome. With samples from 269 children in 29 pools, we were likely underpowered to detect a difference between groups. Indeed, with a post-hoc power calculation we had 60% power to discern a 40% difference in richness and just 18% power to discern a 20% difference. Moreover, the fidelity of the intervention was suboptimal. One of the study intervention wells never hit water, two were functional in the wet season only and one was not functional after three months. Large public health intervention trials are challenging in very resource-limited settings and a more robust durable water improvement intervention may have shown a reduction in viral transmission. Moreover, clean water is not the only viral transmission pathway of interest. This study provides no information on the role of sanitation facilities, poor hygiene, contaminated food products, or limited sterilization during cooking. Finally, the laboratory staff was not masked to treatment allocation of the trial.

In summary, we provide here a description of the enteric virome of East African children. Expanded use of human virome characterization holds promise to measure changes in viral transmissions resulting from natural phenomena or human interventions.

## Supporting information

S1 Table Characteristics of contigs from viruses of unknown tropism. (XLSX)
